# The cost-effectiveness of initiating ranibizumab therapy in eyes with neovascular AMD with good vision: an economic model using real-world outcomes

**DOI:** 10.1136/bmjopen-2014-006535

**Published:** 2015-05-05

**Authors:** Thomas Butt, Aaron Lee, Cecilia Lee, Adnan Tufail

**Affiliations:** 1 Institute of Ophthalmology, University College London, London, UK; 2 Moorfields Eye Hospital, London, UK; 3 University of Washington, Seattle, WA, USA

**Keywords:** ranibizumab, neovascular AMD, cost-effectiveness, electronic medical record

## Abstract

**Objectives:**

To evaluate the cost-effectiveness of immediate treatment with ranibizumab in patients with neovascular age-related macular degeneration (nAMD) with good (better than 6/12) starting visual acuity compared with current UK clinical guidance of waiting until vision falls below 6/12 to begin treatment, using real-world outcomes data.

**Design:**

A patient-level health economic state transition model based on levels of visual acuity in the better seeing eye was constructed to simulate the costs and consequences of treating patients with nAMD with ranibizumab.

**Setting:**

The model took the perspective of the UK National Health Service (NHS).

**Participants:**

The model was populated with real-world outcomes and resource use from a prospective multicentre national nAMD database study containing 92 976 ranibizumab treatment episodes.

**Interventions:**

Two treatment approaches were compared: immediate intervention with 0.5 mg ranibizumab pro re nata, PRN (on detection of nAMD) or delayed intervention (waiting until vision fell to 6/12 before beginning treatment).

**Main outcome measures:**

Quality-adjusted life years (QALYs) for health states and healthcare costs were accrued for each strategy, and an incremental cost-effectiveness ratio (ICER) was calculated. One-way and probabilistic sensitivity analyses were employed to test the uncertainty of the model.

**Results:**

Over a 2-year time horizon, based on 10 000 Monte Carlo simulations, the early treatment arm accumulated 1.59 QALYs and £8469.79 cost. The delayed treatment arm accumulated 1.35 QALYs and £7460.21 cost. The central ICER estimate was £4251.60.

**Conclusions:**

A model based on real-world data is likely to be a realistic reflection of the health gains and resource use of ranibizumab for nAMD in the UK NHS. Initiating treatment immediately with ranibizumab PRN regimen is a cost-effective strategy compared with current guidance of initiating treatment at a level of 6/12 or worse vision.

**Strengths and limitations of this study**

- This is the first cost-effectiveness analysis of initiating ranibizumab treatment of age-related macular degeneration (AMD) in patients with good vision. Immediate treatment of neovascular AMD (nAMD) with ranibizumab in patients with good starting vision (>6/12) is compared with delayed treatment until vision falls below 6/12.
- This model, based on a large real-world data set, is likely to better reflect treatment patterns and outcomes in clinical practice than trial-based models.
- The results suggest that it would be cost-effective to extend the National Institute for Health and Care Excellence (NICE) guidance to treat nAMD immediately.
- The study required some assumptions to be made about changes in vision that occur before patients begin treatment, which we derived from natural history data from the fellow eyes of patients in the electronic medical record (EMR) data set.

## Introduction

Age-related macular degeneration (AMD) is the leading cause of severe visual loss in patients over the age of 50 years in Europe and North America.^1^ ^2^ Neovascular AMD (nAMD) is characterised by choroidal neovascularisation (CNV), which is the growth of abnormal, choroidal blood vessels beneath the macula, which causes severe loss of vision and is responsible for the majority of visual loss due to AMD.^3^ One of the key mediators implicated in the pathogenesis of CNV in nAMD is vascular endothelial growth factor-A (VEGF). Treatments for CNV target VEGF are administered by injection into the vitreous cavity with high binding specificity to VEGF (anti-VEGF agents). These agents are administered by intraocular (intravitreal) injections with repeat injections as necessary depending on the agent.

Intravitreal injection of anti-VEGF drugs such as ranibizumab (Lucentis, Novartis) is an established therapy to treat nAMD and is the most commonly performed retinal procedure in the UK National Health Service (NHS).^4^

The National Institute for Health and Care Excellence (NICE) issued guidance recommending the use of ranibizumab for nAMD England in August 2008, leading to almost exclusive usage of ranibizumab for nAMD in the UK NHS.^5^

Clinical and economic evidence was initially informed by the Anti-vascular endothelial growth factor Antibody for the Treatment of Predominantly Classic Choroidal Neovascularization in Age-related Macular Degeneration (ANCHOR) and Minimally Classic/Occult Trial of the Anti-VEGF Antibody Ranibizumab in the Treatment of Neovascular Age-Related Macular Degeneration (MARINA) studies, which demonstrated that ranibizumab prevents central vision loss and improves mean visual acuity (VA) at 2 years when given at monthly intervals in eyes with subfoveal nAMD.^6^ ^7^

Consistent with these pivotal studies, NICE recommended that ranibizumab for nAMD should be funded in eyes presenting with VAs between 6/12 and 6/96, which parallels the entry criteria of the pivotal studies. Owing to the trials’ exclusion criteria, no direct evidence exists from phase 3 randomised controlled clinical trials to assess the clinical effectiveness and cost-effectiveness of treating patients presenting with early lesions resulting in vision better than 6/12.

However, patients have been presenting with nAMD to treating centres with better visions since NICE initially supported ranibizumab reimbursement on the NHS in 2008. Current guidance is to wait until vision worsens to below 6/12 before treating. Our group has previously shown that if ranibizumab therapy is initiated in eyes with good visual acuities, the treated eye is more likely to maintain good vision,^8^ and this is consistent with the indirect evidence from the pivotal trials that eyes are more likely to maintain vision than recover lost vision at initiation of treatment.^6^ ^7^

The purpose of this work is to evaluate whether immediate intervention with ranibizumab in the better seeing eye of patients presenting with nAMD with good vision is cost-effective compared with the delayed intervention approach that is currently recommended.

A health economic model with health states based on levels of VA in the better seeing eye was developed. The intervention considered is the initiation of ranibizumab (0.5 mg) treatment using three loading injections+a pro re nata (PRN) protocol for patients with a confirmed diagnosis of nAMD and vision better than 6/12: *immediate treatment*. The comparator is the current standard of care for patients with nAMD, which is no treatment for patients with a confirmed diagnosis of nAMD with vision better than 6/12 and treatment with ranibizumab using three loading injections of ranibizumab at approximately monthly intervals followed by a PRN (3 loading injections+PRN) protocol when vision falls below 6/12: *delayed treatment* (current NHS practice). Effectiveness and resource use was derived from real-life outcomes from treated and untreated (fellow) eyes in 14 centres using ranibizumab for AMD in the UK.^8^

This analysis is the first to assess the cost-effectiveness of treating VA better than 6/12 in nAMD compared with treating only when vision is worse than 6/12 with ranibizumab. Furthermore, the work demonstrates how real-world outcomes and resource use associated with the use of ranibizumab therapy may be used to assess the cost-effectiveness of treating nAMD. These results may be more generalisable to routine clinical practice than models based on randomised controlled trial (RCT) data, and therefore more appropriate to assess the cost-effectiveness of routine use treatment protocol in the NHS.

## Methods

### Model structure

**Figure 1:**
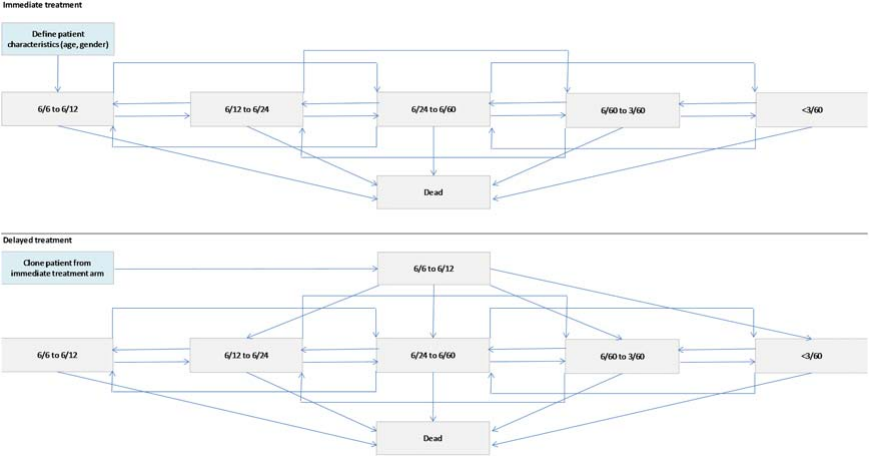
Model structure.

A Markov patient-level simulation model was developed with an initial 3-month cycle followed by monthly cycles. The model consisted of six health states: five health states defined by declining VA ranging from 6/12 or better (least severe) to less than 3/60 (most severe), and an additional absorbing state, death, which was accessible from all levels of vision (figure 1). This model structure was consistent with the model developed by the Evidence Review Group (ERG) in the original NICE appraisal of ranibizumab for nAMD.^5^

On entering the model, a patient was assigned an age and gender based on the distribution of these characteristics among patients with a starting vision of better than 6/12 in the data set.

For *immediate treatment*, a patient was simulated to be treated straightaway on confirmed diagnosis of nAMD with three initial monthly ranibizumab injections followed by PRN for 2 years. For *delayed treatment*, a patient was assigned a time from diagnosis to vision falling below 6/12. In the initial period (>6/12), a patient received no treatment. After reaching 6/12, treatment began and a patient progressed to a state of vision assigned according to a distribution based on the visions of patients beginning treatment in the data set (ie, many eyes with nAMD will initially present with a vision in the NICE guidance allowing immediate treatment but the vision may be any value between 6/12 and 6/96, and not just 6/12). A patient was then treated with three initial monthly ranibizumab injections followed by PRN and continued through the model for 2 years including the starting delay. The simulation was run for 10 000 patients.

### Perspective

The perspective of the model was the UK NHS and Personal Social Services (PSS) as recommended in the NICE Guide to the Methods of Technology Appraisal reference case.^8^ The model had a 2-year time horizon, which represented the time horizon used in pivotal trials. Owing to the short time horizon, costs and benefits were not discounted.

### Transition probabilities

Transition matrices were calculated from the electronic medical record (EMR) data set (table 1). For treatment, transitions were calculated from visual acuities recorded for treated eyes. For no treatment of eyes better than 6/12, transitions were calculated from visual acuities recorded for fellow (untreated) eyes.

**Table 1:** Transition probabilities

Immediate treatment						
		To state				
		6/6 to >6/12	6/12 to 6/24	6/24 to 6/60	6/60 to 3/60	<3/60
*First 3 months (months 0–2), probability for 3-month cycle*						
From state	6/6 to >6/12	0.7240	0.2222	0.0335	0.0108	0.0096
*Remainder of 2 years (months 3–24), probability for 1-month cycle*						
From state	6/6 to >6/12	0.8778	0.1163	0.0046	0.0006	0.0008
	6/12 to 6/24	0.2937	0.6243	0.0783	0.0032	0.0005
	6/24 to 6/60	0.0359	0.2355	0.6747	0.0479	0.0060
	6/60 to 3/60	0.0219	0.0146	0.1533	0.7007	0.1095
	<3/60	0.0588	0.0147	0.0147	0.2059	0.7059

Delayed treatment						
		To state				
		6/6 to >6/12	6/12 to 6/24	6/24 to 6/60	6/60 to 3/60	<3/60
*First 3 months (months after drop to state 2), probability for 3-month cycle*						
From state	6/6 to >6/12	0.0	0.0	0.0	0.0	0.0
	6/12 to 6/24	0.3300	0.4993	0.1506	0.0139	0.0062
	6/24 to 6/60	0.0699	0.3049	0.4923	0.1057	0.0272
	6/60 to 3/60	0.0157	0.0927	0.3795	0.4123	0.0999
	<3/60	0.0203	0.0541	0.2432	0.4257	0.2568
*Remainder of 2 years (+3 from months after reaching state 2), probability for 1-month cycle*						
From state	6/6 to >6/12	0.7366	0.2408	0.0139	0.0026	0.0062
	6/12 to 6/24	0.1433	0.7161	0.1341	0.0054	0.0011
	6/24 to 6/60	0.0081	0.1414	0.7369	0.1068	0.0068
	6/60 to 3/60	0.0047	0.0093	0.2018	0.7045	0.0797
	<3/60	0.0380	0.0087	0.0459	0.2985	0.6089

In the immediate treatment arm, all patients began in state >6/12 with a 3-month loading dose cycle. Patients then received ranibizumab PRN with monthly transitions for the remainder of the 2 years.

For the delayed treatment arm, patients followed a time-to-event survival curve to define the time in state >6/12 before dropping below 6/12 and beginning treatment. Once their vision dropped below 6/12, they entered the 3-month loading dose cycle in the following distribution (state 1: 0, state 2: 0.434484, state 3: 0.3891544, state 4: 0.1456472, state 5: 0.0307501 (based on the distribution of patients beginning treatment in the data set)). Patients then received ranibizumab PRN with monthly transitions for the remainder of the 2 years.

### Utility

Benefits were measured in quality-adjusted life years (QALYs). VA was converted to utility for the calculation of QALYs using Brown *et al*,^9^ which elicited utilities in 80 patients with AMD using the time trade-off method and grouped these by the VA health states defined in the model. The health state utility values used in the model are reported in table 2^10^ and are consistent with those applied to the model used by the ERG in the original NICE appraisal of ranibizumab for nAMD.^5^

**Table 2:** Utility values for model health states

Visual acuity	Utility, mean (SD)
From 6/6 to 6/12	0.89 (0.16)
6/12 to 6/24	0.81 (0.20)
6/24 to 6/60	0.57 (0.17)
6/60 to 3/60	0.52 (0.24)
<3/60	0.40 (0.12)

*Source*: Brown *et al.*^9^

### Cost

Resource use and costs were applied to reflect UK clinical practice. Resource use consisted of monthly assessment visits and ranibizumab injection. On initiation of treatment, patients received three loading doses of ranibizumab as recommended by clinical guidance followed by PRN injections at a frequency calculated from the data set.

UK unit costs were assigned for a cost year of 2012. A cost of ranibizumab of £742.17 per injection, an assessment cost of £255.00 and a monitoring cost of £60.00 was used.^11^ ^12^ These costs were consistent with the NICE costing template for aflibercept (July 2013).

### Sensitivity analysis

Appropriate probability functions were fitted to model parameters to incorporate uncertainty. Probabilistic sensitivity analysis was performed using a Monte Carlo simulation to randomly sample each parameter. Utilities were characterised by a β distribution, with α and β parameters defined by the means and SDs of the utilities. Costs were characterised by a γ distribution with α and β parameters defined by the means and SDs of the costs. SDs were not available for costs, therefore they were assumed to be 10% of the mean in line with recommended practice for health economic models. Transition probabilities were characterised by a Dirichlet distribution. A cost-effectiveness acceptability curve (CEAC) was constructed to represent the probability of the treatment proving cost-effective at a given value of health effect. One-way sensitivity analysis was employed to test structural uncertainty within the model.

### EMR data set

We have previously described the methodology of obtaining the large data set of 92 976 ranibizumab injections,^10^ which covered data from the approval of ranibizumab in August 2008 until April 2012. In brief, 14 NHS hospitals that deliver ranibizumab AMD treatment services in England and Northern Ireland submitted data to this study. Each site is the only NHS provider of nAMD care to their local population and very few patients switch between providers. Following NICE approval for the use of ranibizumab for nAMD in the NHS in August 2008, all sites used this drug almost exclusively. The lead clinician and Caldicott Guardian (who oversees data protection) at each centre gave written approval for the data extraction. Patient identifiers were completely stripped out, and site and clinician data were pseudo-anonymised, and on this basis an ethics committee determined that formal ethics approval was not required. This study was conducted in accordance with the declaration of Helsinki and the UK's Data Protection Act.

The 14 sites entered their first treatment episodes into the EMR system during the following years: 2006 (n=2 sites), 2007 (n=5), 2008 (n=4), 2009 (n=1) and 2010 (n=2). The first recorded ranibizumab injection was dated November 2006.

Over the period of data collection, anti-VEGF treatment was performed in 13 774 patients, of whom 2639 received anti-VEGF for reasons other than nAMD or received bevacizumab. Thus, this study analyses data on 12 951 eyes of 11 135 patients who received a total of 92 976 ranibizumab injections during 317 371 clinic visits at 14 UK hospitals. In total, 16.3% (n=1816) of these patients required treatment to both eyes during the follow-up period. The demographics of the patients included have previously been described and are summarised in table 3.^10^

**Table 3:** Demographic details of patients used to develop model

Variable	Male (n=4071)	Female (n=7062)	Not specified (n=1)	Total (n=11 135)
Age (years)				
Mean	78.8	80.1	79	79.7
Median	80	81	79	81
IQR	74–84	76–86	–	75–85
Range	55–103	55–108	–	55–108

‘Best-measured VA’ was the best VA with refraction or habitual correction and/or pinhole as measured on an Early Treatment Diabetic Retinopathy Study (ETDRS) chart and expressed as ETDRS letters and LogMAR vision in this study.

### Missing data

For patients whose data were not available for a particular visit or had been lost to follow-up, no missing value substitutions were performed. The only exception to this rule was baseline VA, as some treatment centres brought patients back for a two stop service—assessment on first visit followed by injection on second visit, and did not repeat VA measurements on the date of the first injection (n=1670), which was always performed within 3 weeks. This was therefore not missing data per se but reflects variation in treatment delivery. In the model, we assumed no differences between centres for resource use associated with service delivery.

## Results

The central ICER estimate from PSA was £4251.60 per QALY for immediate intervention compared with delayed intervention (table 4). In the immediate intervention group, patients accumulated on average 1.59 QALYs and £8469.79 costs over 2 years versus 1.35 QALYs and £7460.21 costs in the delayed intervention group.

**Table 4:** Central cost-effectiveness results: average of Monte Carlo analysis

	Comparator (delayed intervention)	Intervention (immediate intervention)	Incremental
Cost (£)	7460.21	8469.79	1009.58
QALYs	1.35	1.59	0.24
ICER (£)			4251.60

ICER, incremental cost-effectiveness ratio; QALY, quality-adjusted life year.

**Figure 2:**
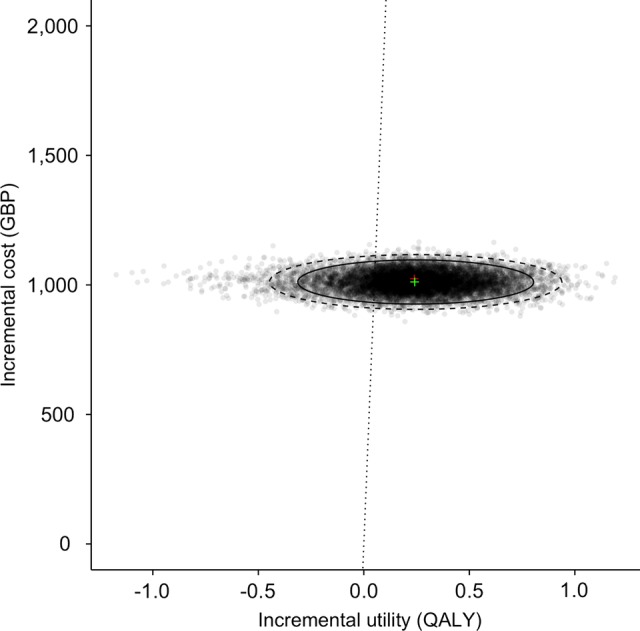
Cost-effectiveness plane (GBP, British Pounds; QALY, quality-adjusted life year).

**Figure 3:**
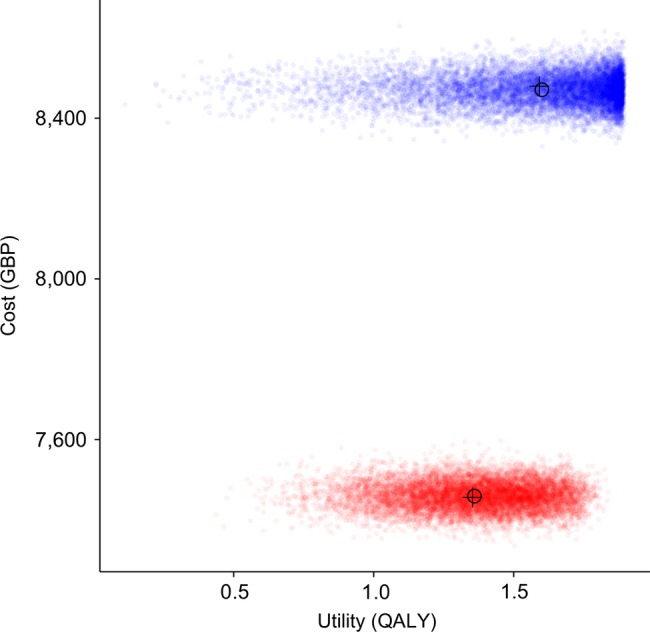
Costs and QALYs accumulated over 2 years by patients treated with ranibizumab according to current NHS practice (red) and with early intervention (blue). GBP, British Pounds; NHS, National Health Service; QALY, quality-adjusted life year.

Figure 2 shows the cost-effectiveness plane with 10 000 simulations. The majority of the distributions are located to the lower right of a £20 000 willingness to pay threshold. The results are disaggregated into the incremental cost per QALY of immediate intervention and delayed intervention in figure 3.

**Figure 4:**
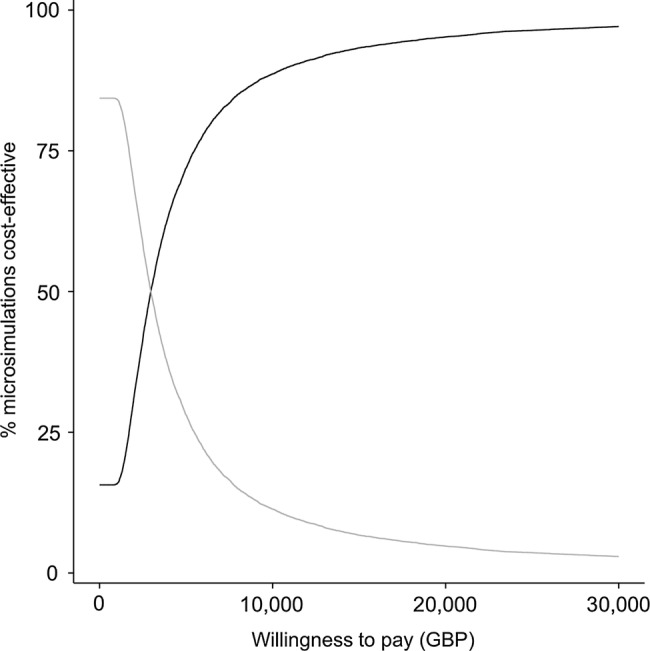
Cost-effectiveness acceptability curve of immediate treatment of nAMD with ranibizumab (dark grey) compared with current NHS practice of delayed treatment (light grey). GBP, British Pounds; nAMD, neovascular age-related macular degeneration; NHS, National Health Service.

Figure 4 shows the CEAC. Immediate treatment has a 50% chance of being cost-effective compared with current treatment practice if the NHS were willing to pay £4251.60 per QALY. At a willingness to pay threshold of £20 000 per QALY, immediate treatment has a >90% chance of being cost-effective.

One-way sensitivity analysis is reported in table 5. The model was sensitive to time horizon. Running the model for 5 years rather than 2 resulted in a lower ICER of £1773.21 (58% lower than the base case). Over a longer time horizon, the early intervention arm accumulated more QALYs for a marginally higher cost than the delayed intervention arm. A younger starting age had a marginal impact on the ICER, with a starting age of 60 years generating an ICER of £3909.36 (8% lower than the base case). Including only drug cost (no visit cost) led to an ICER of £3697.82 (13% lower than the base case). The ICER was also impacted by the choice of health state utility values. Using values elicited by Brown *et al* using the standard gamble technique generated an ICER of £5126.51 (21% higher than the base case using time trade-off values from the same source).

**Table 5:** One-way sensitivity analysis

	Base case	Sensitivity	Cost (£)			QALY			ICER	Change in ICER (%)
Parameter			Comparator	Intervention	Difference	Comparator	Intervention	Difference		
Base case	–	–	7460.21	8469.79	1009.58	1.35	1.59	0.24	4251.60	–
Utility	Brown *et al* TTO	Brown *et al* SG	7460.21	8469.79	1009.58	1.54	1.73	0.19	5126.51	+21
Cost	Drug and visit	Drug only	6232.08	7110.14	878.06	1.35	1.59	0.24	3697.82	–13
Time horizon	2 years	5 years	15 000.16	15 998.43	998.27	3.01	3.57	0.56	1773.21	–58
Starting age	78.8*	60†	7771.01	8768.44	997.43	1.42	1.67	0.25	3909.36	–8

*Source*: Brown *et al*.^9^

*Distribution defined by characteristics of data set.

†Fixed starting age for cohort.

ICER, incremental cost-effectiveness ratio; SG, standard gamble; TTO, time trade-off; QALY, quality-adjusted life year.

## Discussion

Immediate intervention in nAMD is likely to be a cost-effective strategy. Over 2 years, patients received an average of one more injection and gained 0.24 QALYs compared with current practice of delayed intervention.

The ICER of £4251.60 of treating early versus current treatment practice is substantially below a threshold of £20 000 per QALY, which is often considered the NHS’s willingness to pay for health gain.^8^

This is, to our knowledge, the first assessment of the cost-effectiveness of treating patients with vision better than 6/12. We believe that the recommendation of treating patients with vision worse than 6/12 was based on the absence of evidence in patients with better vision due to the exclusion criteria in clinical trials of ranibizumab. Therefore, NICE currently does not recommend funding for eyes with good VA, which may result in some patients having to drop below 6/12 to initiate therapy. From a patient perspective, what is more important is maintaining a good functional visual state that allows continuing to be able to read and drive; waiting until vision falls below 6/12 can be anxiety provoking and delayed treatment can result in worse clinical outcome.^13^ This paper provides evidence that early ranibizumab treatment is associated with a small incremental cost per QALY within the range that the NHS is typically willing to pay for health gain.^8^

The database shows that patients are presenting at centres with AMD with good starting vision. In order to determine the budget impact of extending ranibizumab treatment to visions better than 6/12, the full incidence of early AMD in the population, and the availability and effectiveness of screening, need to be examined. Rates of clinical presentation and screening effectiveness were identified as major areas of uncertainty in a model assessing the cost-utility of a screening programme for early AMD.^14^

It is also possible that earlier treatment could have a different effect on vision. For example, treating AMD at an earlier stage when lesions are smaller could mean that fewer injections may be needed to maintain vision. Further work investigating the cost to the healthcare system of earlier detection and treatment would be valuable future research.

As the first assessment of the cost-effectiveness of treating a broader range of visual acuities with ranibizumab, the results cannot be directly compared with other models. In NICE's economic evaluation of ranibizumab for AMD, the assessment group used a similar state transition model.^5^ The base case ICERs over a 10-year time horizon for predominantly classic lesions were £15 638 per QALY gained compared with photodynamic therapy with verteporfin, and £11 412 per QALY gained compared with best supportive care. For minimally classic lesions and occult lesions, assuming 2 years of treatment, the ICER was £25 098 per QALY gained compared with best supportive care. In terms of clinical effectiveness, VA outcomes from the database previously reported that outcomes do not match the results achieved in most randomised trials, but they were delivered with substantially fewer injections and hospital visits.^8^

This paper synthesises outcomes from routine NHS treatment, which is likely to better reflect real-world effectiveness and resource use than RCT evidence. Beyond the limited range of visual acuities included in pivotal trials, the use of RCT data for assessing cost-effectiveness suffers from limitations of inclusion/exclusion criteria and protocol-driven treatment patterns. Thus, the outcomes and treatment practices derived from RCT data may not reflect today's clinical practice. By contrast, the use of real-world data requires robust methods to deal with non-standardised aspects such as missing data.

There are a number of limitations to this study. First, the study required some assumptions to be made about changes in vision that occur between patients not being treated, which we derived from natural history data, and patients beginning treatment, which we derived from the EMR data set. Once the delayed treatment group initiates therapy, they immediately fall to the starting VA of any person starting on treatment. Meaning that once they fall below the 6/12 line their VA state changes to match the distribution of starting VA in the data set of anyone beginning treatment. We believe that this is realistic in clinical practice, since most lesions are likely to go through subtle changes that can be seen clinically before the patient notices them or before the lesions qualify for treatment. The survival curve on which the model is based uses the fellow eye's structural optical coherence tomography data in the EMR data set. Once the lesion causes the vision to fall below 6/12, patients could realistically end up with any possible vision, clinically.

Second, due to the limited number of VA states, a significant number of patients in the treat-early group remain in the best VA state for the lifetime of the model. Such a situation is perhaps not surprising: Ranibizumab treatment is generally associated with a maintenance of vision rather than an improvement (recovery of lost vision due to nAMD). Therefore, in the model initiating treatment early, patients maintained a better VA state and accumulated more QALYs.

In summary, our study provides a real-world data based model demonstrating that early ranibizumab intervention is associated with an acceptable incremental cost that is well within the NHS acceptable range to pay for health gain. Thus, the maintenance of better VA in patients who are treated early is not only beneficial clinically but also likely cost-effective. This study may help inform future policy decision regarding the routine treatment with ranibizumab in patients having visual acuities better than 6/12.
